# Acceptability of Telehealth‐Delivered Occupational Therapy Among Individuals With Long COVID Using the Theoretical Framework of Acceptability: A Qualitative Study

**DOI:** 10.1155/ijta/8879520

**Published:** 2025-12-12

**Authors:** Christina Müllenmeister, Gloria Königs, Stephanie Heinemann, Dominik Schröder, Frank Müller, Eva Hummers, Andrea Stölting, Christine Happle, Alexandra Dopfer-Jablonka, Georg Behrens, Ulrike Marotzki, Tim Schmachtenberg

**Affiliations:** ^1^ Department of General Practice, University Medical Center Goettingen, Goettingen, Germany, uni-goettingen.de; ^2^ Faculty of Social Work and Health, Hawk University of Applied Sciences and Arts Hildesheim, Goettingen, Hildesheim, Germany; ^3^ Department of Geriatrics, University Medical Center Goettingen, Goettingen, Germany, uni-goettingen.de; ^4^ Department of Family Medicine, Michigan State University, Grand Rapids, Michigan, USA, msu.edu; ^5^ Department of Rheumatology and Immunology, Hannover Medical School, Hannover, Germany, mh-hannover.de; ^6^ Department of Pediatric Pulmonology, Allergology and Neonatology, Hannover Medical School, Hannover, Germany, mh-hannover.de; ^7^ Biomedical Research in End-Stage and Obstructive Lung Disease (BREATH), Hannover Medical School, Hannover, Germany, mh-hannover.de; ^8^ German Center for Lung Research (DZL), RESIST Cluster of Excellence, Hannover, Germany, dzl.de; ^9^ German Center for Infection Research, Partner Site Hannover-Brunswick, Hannover, Germany, dzif.de

## Abstract

**Background:**

Long COVID still challenges healthcare systems worldwide. Tailored treatments are scarce. In the ErgoLoCo study, we have developed and tested a telehealth‐delivered occupational therapy intervention for people affected by long COVID. Acceptability from both recipients and providers is a prerequisite for implementing such new interventions.

**Aim:**

This study is aimed at exploring the perceptions of people with long COVID and occupational therapists regarding the intervention′s acceptability and telehealth delivery approaches.

**Methods:**

Semistructured interviews were conducted with 13 participants who experience long COVID and received the ErgoLoCo intervention delivered as teletherapy sessions or prerecorded videos. Eight occupational therapists who guided the teletherapy sessions participated in a focus group. Materials were analyzed following qualitative descriptive methods and interpreted using the theoretical framework of acceptability (TFA).

**Results:**

Occupational therapists and long COVID clients considered the occupational therapy approach a positive experience. While all participants in the teletherapy group found the occupational therapy approach helpful in coping with long COVID symptoms and regaining participation in meaningful occupations, perceptions varied in the group supplied with prerecorded videos. Some saw the intervention as helpful, but all emphasized the need for professional support from occupational therapists to use the program more effectively. The occupational therapists emphasized the need to tailor the therapy content to clients′ needs to ensure effective and successful management of occupational challenges.

**Discussion:**

The study highlights telehealth‐delivered occupational therapy′s potential benefits and challenges for individuals with long COVID. It contributes to understanding the challenges and potential of telehealth‐delivered occupational therapy for long COVID rehabilitation. This study′s key finding is the importance of personalized and professionally guided telehealth interventions.

**Trial Registration:** German Clinical Trial Registry identifier DRKS00029990

## 1. Introduction

Long COVID, also known as post‐acute sequelae of SARS‐CoV‐2 infection (PASC), is still challenging healthcare systems worldwide [1, 2]. Affected people may experience various persistent symptoms after an acute COVID‐19 infection, such as fatigue, brain fog, dizziness, shortness of breath, or muscle pain [3–5].

Affected people often struggle to access care, and feasible and tailored rehabilitation strategies and quality of life are urgently needed [6, 7]. The complexity of the syndrome underscores the need to innovate and refine diagnostic and therapeutic modalities to ensure greater precision and improve client care that can address medical challenges with a more focused attitude [8–10].

Occupational therapy is an essential part of the multimodal management of long COVID and is recommended in treatment guidelines [11–13]. Occupational therapy focuses on enabling people to regain autonomy in self‐management and restoring engagement, performance, and participation in meaningful occupations. Meaningful occupations encompass activities people want, need, or are expected to do daily, including work, self‐care, or leisure activities [14–18]. Research indicates that inpatient and outpatient occupational therapy interventions for long COVID clients encompass a variety of strategies to enhance self‐management, functionality, and independence in daily life, which are applicable in the context of long COVID rehabilitation [19–24].

Studies have particularly highlighted the importance of easy access, equity, and minimal client care burden, acknowledging the fluctuation of symptom strengths in long Covid [25, 26]. Telehealth approaches may have advantages as they can be easily accessible to a large number of affected people and should thus be further explored [27–29]. We use the term telehealth as defined by the World Federation of Occupational Therapists (WFOT):’[…] the use of information and communication technologies (ICTs) to deliver health‐related services when the provider and client are in different physical locations’ (p 37) [30]. These services can be synchronous, such as videoconferencing consultations between an occupational therapist and a client, or asynchronous, such as prerecorded instruction via video, e‐mail, photos, and so forth [30]. Recent studies point to telehealth in occupational therapy providing effective models of provision [31–35].

Other authors have demonstrated the potential of telehealth interventions for long COVID clients, for example, through online peer support groups, digital physiotherapy interventions, or programs aimed at improving physical and mental health [36–43]. Despite these promising approaches, to our knowledge, telehealth interventions developed for long COVID in occupational therapy have not been evaluated yet. The acceptability of synchronous versus asynchronous occupational therapy telehealth concepts is a further unstudied topic, but it is important to prevent non‐use and allow a successful implementation [44].

This study is aimed at exploring the intervention′s acceptability by applying the theoretical framework of acceptability (TFA). TFA accounts for both cognitive and emotional responses and thus provides insights into the perceived appropriateness of telehealth‐delivered occupational therapy for both providers and long COVID recipients [45]. This framework facilitates a comprehensive acceptability assessment across prospective, concurrent, and retrospective studies and has been used in various studies [46–48].

## 2. Materials and Methods

### 2.1. Study Setting and Design

In this paper, we report on the results of the process evaluation of the ErgoLoCo project. In ErgoLoCo, a concept for telehealth‐delivered occupational therapy was developed. Feasibility and efficacy were assessed in a randomised controlled pilot trial (pilot‐RCT). Details are outlined in a study protocol [49]. In brief, *n* = 158 participants with long COVID were randomly assigned to (a) a synchronous teletherapy group, (b) an asynchronous video group, or (c) a control group (watch‐and‐wait). Both intervention groups received occupational therapy twice weekly for 12 weeks. Efficacy outcomes have been reported in a separate paper [50].

As part of the process evaluation, we employed a qualitative descriptive design to elucidate individual variations in experiences and outcomes to foster the ongoing refinement of the intervention [51]. We used the qualitative approach to acknowledge that the acceptability of an intervention is subjectively evaluated by those undergoing or guiding the intervention [45]. Therefore, this study is aimed at exploring participants′ and occupational therapists′ perceptions of the program′s acceptability.

### 2.2. The Occupational Therapy Program

The occupational therapy program comprises six modules, each containing four 30‐min therapy sessions. Professional occupational therapists led the teletherapy group, while the video group completed the program independently with video instructions. All participants received a workbook containing session materials and “do@home‐tasks” to reinforce therapy in daily life. The Canadian Occupational Performance Measure (COPM) was used to assess occupational performance and satisfaction before the first session and after the last session. The program was developed by CM, an experienced occupational therapist, based on a literature review and expert input from Germany, Austria, and Switzerland, aims to enhance self‐management and engagement in meaningful activities [49, 52]. Table 1 provides an overview of the program.

**Table 1:** Overview of the occupational therapy program, consisting of 24 therapy sessions, structured in 6 consecutive modules, including four therapy sessions (INT) each; COPM, Canadian Occupational Performance Measure.

**Module**	**Intervention**	**Content**
1 Inventory	INT 1	Entry to OT and identification of occupational concerns arising from Long COVID (COPM)
	INT 2	Analysis of occupational performance problems
	INT 3	Understanding occupational performance problems
	INT 4	Identifying resources to comprehend and recover the client’s occupational repertoire

2 Occupational balance	INT 5	Managing occupational performance challenges by utilizing available resources
	INT 6	Learning to balance occupational requirements
	INT 7	Break‐management
	INT 8	Applying break management in everyday life

3 Manage everyday situations	INT 9	Develop strategies for carrying out meaningful activities and occupations
	INT 10	Implement strategies for carrying out meaningful activities and occupations
	INT 11	Plan and perform complex occupations
	INT 12	Monitoring day‐to‐day management

4 Everyday life training	INT 13	Everyday life training (1)
	INT 14	Everyday life training (2)
	INT 15	Everyday life training (3)
	INT 16	Everyday life training (4)

5 Autonomy in everyday life	INT 17	Coordinator of everyday life (self‐care)
	INT 18	Coordinator of everyday life (productivity)
	INT 19	Coordinator of everyday life (leisure)
	INT 20	Occupational Balance

6 Well‐prepared for the future	INT 21	Monitoring—evaluation of achievements and clarification of further concerns (self‐care)
	INT 22	Monitoring—evaluation of achievements and clarification of further concerns (productivity)
	INT 23	Monitoring—evaluation of achievements and clarification of further concerns (leisure)
	INT 24	Re‐evaluation and completion of the therapy program (COPM follow‐up)

### 2.3. Participants and Recruitment

**Figure 1:**
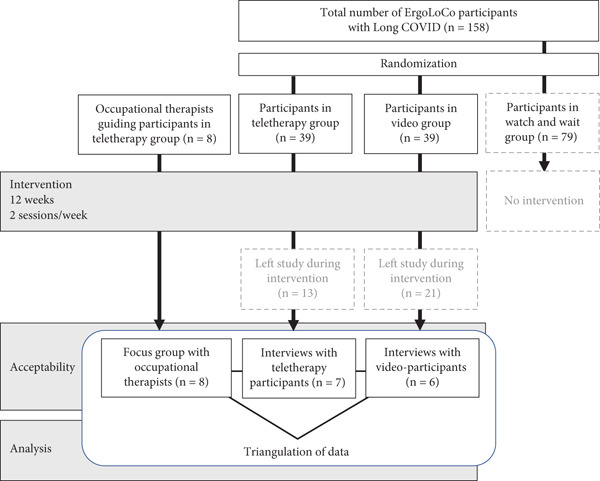
Flowchart illustrating the acceptability evaluation of the occupational therapy program within the ErgoLoCo study.

To assess the program′s acceptability, we purposely selected participants from both intervention groups of the ErgoLoCo study who were willing to share their experiences. Interested individuals contacted the first author by phone or e‐mail and were informed orally and in writing about the study′s content and purpose. Thirteen participants (*n* = 7 teletherapy and *n* = 6 video) consented to individual interviews. To ascertain occupational therapists′ perspectives, all eight therapists who conducted the teletherapy group were invited to a focus group, and all (*n* = 8) participated in an online meeting in May 2023 (see Figure 1).

### 2.4. Data Collection

Between January and June 2023, the first and last authors conducted one‐on‐one online interviews with participants and a focus group with occupational therapists in the German language. We used interviews to gain deeper insights into participants′ experiences and perceptions of acceptability. The semistructured interview guide was based on the eight constructs of the TFA [45, 53]. CM developed the interview guide and refined it iteratively after discussing it with the research team. It included eight questions aligned with the constructs of the TFA (see Table 2). For example, participants were asked to explain what had helped them most to implement therapy content in their everyday lives (self‐efficacy), or describe what they had benefited most from the program (perceived effectiveness). The guide was piloted with one teletherapy participant and further refined. We have included this pilot interview in the analysis (P1). Interviews lasted between 28 and 67 min, and no other persons than the researcher and the participant were present.

**Table 2:** Overview of the TFA constructs used for deductive coding defined by Sekhon et al. (2017).

**Construct/category**	**Description**
Affective attitude	How an individual feels about the intervention
Burden	The perceived amount of effort that is required to participate in the intervention
Ethicality	The extent to which the intervention has a good fit with an individual′s value system
Intervention coherence	The extent to which the participant understands the intervention and how it works
Opportunity costs	The extent to which benefits, profits or values must be given up to engage in the intervention
Perceived effectiveness	The extent to which the intervention is perceived as likely to achieve its purpose
Self‐efficacy	The participant’s confidence that they can perform the behavior(s) required to participate in the intervention

To gather the perceptions of occupational therapists, we conducted one online focus group [54]. Conducted on May 9, 2023, the 90‐min session was moderated by the first author using a moderation guide that was also informed by the TFA and included condensed discussion topics emerging from the interviews with the participants. Discussion topics were for example: feasibility and acceptability of content and the digital nature and the implementation of the program. The last author took notes during the focus group discussion to record salient topics directly and introduce questions into the discussion round that arose due to the content discussed. Interviews and the focus group were audio recorded and conducted using Zoom videoconference software (Zoom Video Communications, Inc., San José, CA, United States). The online setting during interviews and the focus group was chosen to include individuals with mobility constraints due to their health condition, and those who lived at a greater distance from the study site had the opportunity to participate in the study.

### 2.5. Data Analysis

All data were analyzed in German. A professional transcription service transcribed all audio files verbatim following the recommendations by Dresing and Pehl [55]. We anonymized the transcripts to ensure confidentiality and coded them as P1–P13 for Long COVID participants and OT1–OT8 for occupational therapists. CM, a female experienced occupational therapist and TS, a male sociologist and postdoc researcher, conducted the qualitative content analysis as outlined by Kuckartz and Rädiker [56]. This method enabled a systematic and nuanced exploration of experiences.

Both researchers independently read the transcripts, highlighted significant passages, and documented content anomalies. Then, memos outlined evaluation ideas related to the seven acceptability constructs [45]. Consensual coding was employed using a deductive approach. Both authors independently coded one‐third of the material before reaching a consensus on the final code system. Subsequently, a codebook was created based on the TFA constructs, including category definitions and exemplary quotations. Table 3 presents an extract of the codebook. To minimize distortion of the nuances and meanings of the participants′ literal contributions, the third author, a native English speaker, translated the quotes used to present the results in this article into English.

**Table 3:** Extract of the codebook for perceptions on acceptability of the ErgoLoCo program using the theoretical framework of acceptability (Sekhon et al.,2017).

**Code/TFA constructs**	**Description (Sekhon et al., 2017)**	**Illustrative statement participant from video group**	**Illustrative statement** **Participant teletherapy group**	**Illustrative statement** **Occupational therapist**
Intervention coherence	‘The extent to which the participant understands the intervention and how it works.’	‘Because I […] did not do a good job of breaking things down for myself regarding my situation. I think more would be possible in direct contact with an occupational therapist. Because that is something that I cannot manage myself.’ (P9/Video)	‘The therapist explains the Do@Home task and then asks me again: ‘Do you know what you must do?’ I then have the opportunity to ask. It is also described again in the booklet, so if I forget what she told me after the session, I can read it again and return to it. But we always talk about it again at the end of the lesson, and that is very important for me to understand it.’ (P7/Teletherapy)	‘I found that the (program) covered many facets in an occupation‐focused therapy approach. […] Many different areas were addressed, and I felt everyone could find themselves in them. I think each Long COVID client had problems in one of these areas. And then you could do a bit more in that area or expand it a bit and shorten something else or work through it more quickly, so to speak.’ (OT6)

We assigned the entire material to differentiated categories using consensual coding in the second coding process. After creating interim thematic summaries for individual cases, we conducted a category‐based analysis. This phase involved paraphrasing, quantitatively representing, and qualitatively interpreting the statements from participants and occupational therapists [44]. MAXQDA software (Version 20.0.8, VERBI Software GmbH, Berlin, Germany) was used for all coding processes.

### 2.6. Ethical Considerations

The entire ErgoLoCo project was approved by the Institutional Review Boards of Hannover Medical School (9948_BO_K_2021) and the University Medical Center Göttingen (15/8/22 U). All participants and occupational therapists provided written informed consent before the interviews or focus group sessions. Results are presented by Consolidated Criteria for Reporting Qualitative Research (COREQ) [57].

## 3. Results

### 3.1. Demographic Characteristics of the Participants of the Intervention Groups

The participants receiving telehealth‐delivered occupational therapy were aged between 23 and 56 years. Then, 11 out of 14 were female. Only four participants stated that they had prior experience with occupational therapy. While most were familiar with digital devices and online conferencing, online therapy was a new experience for them (see Table 4).

**Table 4:** Characteristics of the participants of the intervention groups.

**Participant**	**Sex**	**Age**	**Working status**	**OT-experiences**	**Experience with digital tools/online therapy**
Teletherapy group					
P1	Male	35	Sick leave since June 2022	Outpatient practice	Video‐conferencing
P2	Female	33	Sick leave since February 2023	None	Not specified
P4	Female	24	Working	None	Online training
P5	Female	29	Working, studying	None	Online training
P7	Female	46	Working	None	Not specified
P10	Female	33	Studying	Outpatient practice	Online training
P12	Male	26	Sick leave since May 2022	None	Video‐conferencing, online therapy
Video group					
P3	Female	35	Sick leave since April 2022	None	Video‐conferencing, Online training
P6	Female	36	Sick leave since September 2022	Inpatient rehabilitation	Use of video‐sharing platforms
P8	Female	56	Sick leave since September 2021	Inpatient rehabilitation	Little experience with digital devices/tools
P9	Female	46	Working	Outpatient practice	Not specified
P11	Female	36	Sick leave since April 2022	None	Video‐conferencing
P13	Female	23	Studying	None	Online training

All participants in the intervention groups experienced persistent symptoms following SARS CoV‐2 infection, lasting from 3 to 11 months, which interfered with daily life to varying degrees. Table 5 outlines functional complaints and occupational performance issues mentioned by the interview participants.

**Table 5:** Participant′s perceptions of long COVID symptoms and occupational problems.

**Participant**	**Duration of long COVID**	**Reported symptoms/functional problems**	**Reported occupational performance problems**
Teletherapy			
P1	11	Brain fog, cognitive impairment, myasthenia, exertional dyspnea	Stair climbing, walking longer distances, reading/understanding texts
P2	36	Fatigue, brain fog, cognitive impairment, perceptual disorders	Showering, cooking, reading books
P4	24	Fatigue, brain fog, cognitive impairment, exertional dyspnea, irritability, melancholy	Cycling, work incapacity
P5	24	Fatigue, brain fog, diminished endurance	Studying, writing, work incapacity
P7	5	Brain fog, cognitive impairment, pain, exertional dyspnea	Climbing stairs, doing sports, maintaining social contacts, household activities
P10	9	Fatigue, brain fog, cognitive impairment, diminished endurance	Taking walks, grocery shopping, reading
P12	10	Fatigue, pain	Cooking, grocery shopping, taking walks
Video			
P3	8	Fatigue, pain, muscle atrophy	Stair climbing, household activities, grocery shopping, child care activities, reading, writing, meeting friends, work incapacity
P6	9	Fatigue, brain fog, cognitive impairment	Reading, meeting friends, preparing meals, doing Yoga
P8	14	Fatigue, pain, exertional dyspnea	Reading, listening to radio news, car driving, household activities, gardening
P9	3	Brain fog, cognitive impairment, pain	Reading, work incapacity, leisure activities
P11	11	Brain fog, cognitive impairment, tachycardia	Meeting people appointments
P13	11	Fatigue, brain fog, vertigo, exertional dyspnea	Grocery shopping, maintaining social contacts

### 3.2. Characteristics of the Occupational Therapists

All occupational therapists in the focus group discussion were female and between 25 and 48 years old. As part of the intervention, they provided occupational therapy using teletherapy following the ErgoLoCo concept for one to eight participants. Occupational therapists had between 2 and 23 years of working experience, and more than half of them had an academic degree in occupational therapy. While five therapists had previous experience with teletherapy, three were new to the online‐based treatment setting. Table 6 provides a summary of the occupational therapists′ characteristics.

**Table 6:** Characteristics of the occupational therapists.

**Occupational therapist**	**Sex**	**Age**	**Degree**	**Working experience (years)**	**Experience in long COVID treatment**	**Number of study**—**clients**	**Experiences in telehealth**
OT1	Female	38	Vocational training	15	No	5	No
OT2	Female	45	Bachelor′s degree	21	No	1	Yes
OT3	Female	25	Bachelor′s degree	4	Yes	8	Yes
OT4	Female	29	Bachelor′s degree	5	Yes	2	Yes
OT5	Female	28	Master′s degree	6	Yes	4	Yes
OT6	Female	26	Vocational training	2	Yes	6	No
OT7	Female	48	Vocational training	23	Yes	3	Yes
OT8	Female	26	Bachelor′s degree	3	Yes	4	No

### 3.3. Perceptions on the Acceptability of the ErgoLoCo Concept

#### 3.3.1. Affective Attitude

Most of the participants in both telehealth groups expressed gratitude for the opportunity to participate in a therapy program for Long COVID rehabilitation. This was due to the limited opportunities for long haulers at the time.

*“What touched me very, very, very much right from the start was that there are people who take us Long COVID patients seriously and consider us worthy of research and that people are looking into it: How can we help you?” (P2/Teletherapy)*

Similarly, the occupational therapists who guided participants from the teletherapy group through the program articulated their satisfaction in having the opportunity to base their work on a concept specifically developed for treating long COVID.

*“I also think it′s great that there is such a specific occupational therapy program for Long COVID sufferers or that it has been tested.” (OT5)*

Participants in the video group added that they felt taken seriously, particularly through the empathetic speaking style and the way of leading recipients through the intervention in the videos.

*“So I feel like I′m taken seriously in the way I′m guided, so to speak, in the way I′m spoken to in an empathetic way.” (P3/Video)*

Only one person expressed negative emotions toward the program. It reminded her of psychotherapy and produced frustration and feelings of either over or under‐challenged by the tasks, resulting in an overall negative attitude towards the program.

*“It reminded me a lot of psychotherapy, and I′ve had some pretty bad experiences with psychotherapy. So, of course, it was really stupid; sometimes, I thought I was too stupid, and sometimes, I thought I was too intelligent. Those were the emotional swings, and it was just annoying.” (P9/Video)*

#### 3.3.2. Burden

Participants in the teletherapy group and the occupational therapists described not having to make much effort to participate in or guide the therapy sessions.

*“I′m glad I don′t have to go somewhere else, so it fits very well into my everyday life. This means I can also integrate it with long COVID [symptoms].” (P4/Teletherapy)*

An occupational therapist mentioned that only technical issues with the conference software occasionally required finding quick and pragmatic solutions so that therapy could take place.

*“I had little influence on the technical difficulties when the sound didn′t work or the camera or you couldn′t dial in at all. Of course, that certainly depends on the platform. I dealt with it flexibly and sometimes used a different platform to be able to do it at all.” (OT5)*

Participants in the video group confirmed the low level of effort required to participate in the therapy sessions due to the flexible access to the videos on days, times, and frequency of their choice. While this flexibility was perceived as convenient, participants also described an emerging pressure if they could not keep up with watching and working through the material in the planned 2‐week module.

*“I wanted to plan to watch things again later, but then the new videos come in every time. And then it puts a bit of pressure on me in that sense. […] On the one hand, it′s practical that the videos are flexible. On the other hand, I personally also find it very difficult to stick to these times [twice a week] if I′m feeling extremely poorly on a particular day.” (P6/Video)*

Participants underwent a learning process to determine when and how much effort to put into watching videos independently. They noted the need to establish their own pace and structure for therapy.

*“So at the beginning, I said, well, do the video, sit down straight away, do the workbook. Now I see how much strength and concentration I have left after the video, and sometimes, if I can′t do it, I just do the task later. The good thing is that you can watch the videos several times if you′ve forgotten something or can′t remember something.” (P11/Video)*

Participants in the teletherapy group reported that they negotiated such adaptations with their occupational therapists and tailored the content to their concerns and health status.

*“For example, we′ve now adapted the weekly plan to suit me because I′m not in a position to draw up a plan like that at the moment due to the severity of my condition. Instead, I have to look individually every single day to see what I have available and what effort I can put in.” (P2/Teletherapy)*

#### 3.3.3. Ethicality

The participants of both intervention groups mentioned that they felt valued and accepted as individuals with a novel health condition that is not clearly defined and not yet fully socially accepted or medically understood. For example, one participant in the video group stated as follows:

*“As far as Long COVID is concerned, you feel accepted, which is generally not the case in society or with other doctors. And you also feel understood.” (P3/Video)*

Occupational therapists emphasized that they felt valued for contributing their differentiated occupation‐focused expertise to the therapy process, even though the program was standardized and there was little room for deviation from its basic content to ensure comparability with the video group.

*“Before we started with the intervention, I was very unsure or wondered how it could be implemented and how we could work according to the manual if we wanted to work in a client-centred and individual way. However, I was also pleasantly surprised by the content, which always had such a connection to the COPM at the beginning and then also in the course with the formulation of goals and the evaluation, which I found very nice. But I always knew there was a manual and that I had to stick to it. So, somehow, you automatically respond to individual needs and concerns. And sometimes it was difficult for me to reconcile that with what had to be done in the unit.” (OT5)*

#### 3.3.4. Intervention Coherence

The purpose of the ErgoLoCo concept was to enable participants to regain better performance and participation in meaningful occupations and thus improve self‐management of Long COVID symptoms. Occupational therapists echoed this aim in emphasizing the program′s specific focus on everyday occupations while employing flexibility to address a diverse group of persons with various needs.

*“I found that the (program) covered many facets in an occupation-focused therapy approach. […] Many different areas were addressed, and I felt everyone could find themselves in them. I think each Long COVID client had problems in one of these areas. And then you could do a bit more in that area or expand it a bit and shorten something else or work through it more quickly, so to speak.” (OT6)*

Most participants could grasp the structure of the therapy modules and their contents. P3 stated: *“I think it builds on each other very well” (P3/Teletherapy).* Others acknowledged the importance of continuity in pursuing individual therapy goals set at the beginning of the therapy.

*“With these therapy goals that you set yourself at the beginning, you can always look very specifically at what I can realize.” (P4/Teletherapy)*

One participant from the video group expressed confusion about tasks within the modules, and she struggled to understand their purpose. She also experienced frustration with goal setting, specifically with the suggestion to break her large recovery goal into smaller goals for managing long COVID.

*“I was also extremely annoyed by (…) the goals. I have one goal right at the back: to get better. Everything in me refuses to set myself short-term goals. I don′t do that.” (P9/Video)*

Another person added that it was hard for her to relate the content to her situation and that she would have perceived professional support from time to time as helpful in following the intervention.

*“Because I […] didn′t do a good job of breaking things down for myself regarding my situation. I think more would be possible in direct contact with an occupational therapist. Because that′s something that I can′t manage myself.” (P9/Video)*

A participant from the teletherapy group shared a contrary experience but emphasized that continuous support from the occupational therapist enhanced her understanding of the function of different tasks and how to apply them between therapy sessions.

*“The therapist explains the Do@Home task and then asks me again: ‘Do you know what you must do?’ I then have the opportunity to ask. It′s also described again in the booklet, so if I forget what she told me after the session, I can read it again and return to it. But we always talk about it again at the end of the lesson, and that′s very important for me to understand it.” (P7/Teletherapy)*

#### 3.3.5. Opportunity Costs

Although the therapy′s online format saved participants time and energy due to remote participation, some reported that their engagement restricted them from carrying out other activities or resulted in changes in their usual schedules. For instance, a video‐group participant found the treatment so intense that she could not pursue other activities afterwards.

*“So [the therapy program] is very exhausting. […] And afterwards […] I also need time to rest again. I can′t do anything new immediately, and then I don′t read a book, do other things, or make phone calls.” (P6/Video)*

One participant in the teletherapy group also mentioned that occupational therapy overlapped with an activity that she usually does regularly at this time and had to pause it until she finished her participation in the therapy program.

*“On Mondays, when I have occupational therapy, I would like to meditate, but I can′t do that either. I′ll do that again when I′m finished.” (P4/Teletherapy)*

Occupational therapists mainly valued the synchronous telehealth format. Having a clear guiding treatment concept was perceived as beneficial and time‐saving. The option of organizing therapy sessions more flexibly and remotely from places other than their therapy practices was perceived as an advantage.

*“It was very beneficial for me. I have children of primary school age, and it was very easy to plan the family schedule so that I could still look after my children.” (OT 10)*

#### 3.3.6. Perceived Effectiveness

Some participants mentioned that participating in the program was perceived as a way out of helplessness.

*“That you no longer have this helplessness, this ‘what do I do now?’ and nobody can tell you. ‘Take a rest. Don′t go beyond your limits.’ Yes, where is my limit? ‘Yes, that′s individual.’ That′s what the doctors say, and then you′re left standing there. What is ‘individual’? And, as I said, this has been worked out very well in the program.” (P7/Teletherapy)*

Participants of both groups described that they could partly achieve the goals they had formulated through program guidance at the beginning of the therapy.

*“I had set myself the goal of reading and understanding texts again. I can manage to read a story to my son again. That was definitely worse at the beginning. And I now try to read very small magazine articles myself to understand them. That has already gotten better. And I would say that I′ve achieved part of my goal.” (P3/Video)*

Even if participants did not achieve their full goals, some reported feeling they were progressing towards them by applying strategies such as pacing that they had learned through the program, even in small steps.

*“It′s already the case now that […] through pacing and by carrying out activities consciously and more slowly, I manage to collect small experiences of success […] and I′ll say five steps more […], or yes, the two minutes that I can walk longer, these are experiences of success.” (P1/Teletherapy)*

Concerning how the occupational therapists perceived the achievement of occupational therapy‐specific outcomes, some mentioned before the evaluation phase that they were unsure whether the program could work in an online‐based format. One occupational therapist narrated:

*“I remember it was a huge question mark for all of us at the beginning when we thought: Occupational therapy online? Is that possible? Until we realized all the things you can do online.” (OT6)*

She shared that she experienced it as *“[…] an absolutely great opportunity to bring occupational therapy directly into the home environment and pick people up right where they are.” (OT6).*

Another occupational therapist emphasized her perception of a good fit between Long COVID and occupational therapy due to the profession′s occupation‐focused lens.

*“Exactly, the specific focus on occupation, […] I believe the Long-COVID condition cries out for occupational therapy because it′s about using skills to get back into doing. And I thought the program was just super appropriate and modern.” (OT2)*

They perceived that it was beneficial that the program left room for adaptation, and they could adapt the therapy content with the participants to better suit their everyday situation and the burden of their illness. OT1 explained this using the example of two severely affected participants to whom she provided therapy:

*“For two of my clients with a relatively severe Long COVID course, the issue was that two weekly appointments were difficult for them to organize. And then the question was: What′s the best way to adapt it now? […] Exactly, where we then looked: How can we perhaps place a certain focus and leave out certain parts that are now also less relevant to their everyday lives?” (OT1)*

A participant from the teletherapy group confirmed that.

*“And that′s when we adjusted our goals. For example, we said: OK, cooking is really important to me because I enjoy it and because it makes me feel great when I make delicious food and can then eat it. I think that′s great. And then we focused on that. […]. And then we said: OK, my goal now is not to do more sport, but to break down the showering activity so that I can shower without completely collapsing afterwards. And we actually managed to do that.” (P2/Teletherapy)*

An occupational therapist found the program effective. She noticed that many participants reported that, after some time, they were able to integrate the tasks they had worked on in the therapy into their daily routine and thus eventually benefited from the program.

*“Then they said: “Of course I′ll continue with my weekly break plan and my stress chart too.” Of course, I personally realized that they had applied this and will continue to use it.” (OT4)*

#### 3.3.7. Self‐Efficacy

Participants were consistent in their reports of their confidence in attending both telehealth formats. Besides one participant (P8/Video), all had experiences with the use of digital devices such as personal computers, laptops, tablets, or smartphones that were needed to attend therapy sessions via videoconferencing software or watching a video on a streaming platform.

*“I was already trained in that respect. It wasn′t a new situation […], online really wasn′t a problem for me.” (T11/Video)*

Participants of both groups perceived barriers mostly related to independently managing the complexity of tasks and material, such as watching two videos a week in the video group or working through the do@home tasks on top of their everyday responsibilities outside of therapy.

*“Then I have this do@home task, like writing a weekly plan or something, and then I find it really hard to stick to it when I have ten other things to do and then I′m so exhausted and forget about it in the evening. I find it very, very complex.” (T4/Teletherapy)*

Participants in the video group also mentioned that it was a challenge to keep motivating themselves without any professional or peer support.

*“I just found it very difficult to motivate myself all the time, especially over the twelve-week period. Yes, motivating myself and sitting down again and again.” (T13/Video)*

Otherwise, the participants in both groups and the occupational therapists indicated that they felt sufficiently informed and equipped with the necessary resources and media to perform the therapy effectively.

*“Overall, I think it works quite well. Because you have the booklets and I also have them digitally, I think you have everything you need. And with the video conferences, I think it all works in a very relaxed way.” (P5/Teletherapy)*

## 4. Discussion

This study investigated the acceptability of a telehealth‐delivered occupational therapy concept among individuals experiencing Long COVID and occupational therapists. We utilized the TFA as a conceptual framework to analyze their perceptions [45]. Deductive analysis using the TFA revealed three main aspects of therapy acceptance by participants and occupational therapists: the program′s specific focus on Long COVID, its digital accessibility, and the presence or absence of professional guidance and support from occupational therapists.

### 4.1. Long COVID‐Specific Focus of the Program

The participants highlighted the program′s focus on long COVID, contributing to their acceptance of the intervention. They valued the official recognition of long COVID syndrome and the development of the new occupational therapy intervention, seeing it as a recognition of the burden of the occupational challenges they face in daily life. These perceptions contrast with previous studies where individuals with long COVID often felt misunderstood and neglected by healthcare professionals and policymakers [58–60].

Although studies emphasized the necessity for particular interventions for Long COVID [8, 9], most treatment approaches derive from interventions used for conditions with similar symptoms that are, however, often not tailored to address long COVID‐specific occupational constraints [20, 61, 62]. While some studies have included self‐management or mindfulness exercises in Long COVID rehabilitation, our occupational therapy program uniquely focuses on addressing the individual occupational concerns arising from long COVID symptoms.

Newer standards in occupational therapy practice have shifted towards integrating everyday occupations and collaboratively setting goals with clients to enhance occupational performance, participation, and well‐being [16, 63]. Ensuring quality services through evidence‐based and comprehensive interventions is a major concern of the profession [48]. Standardized approaches may not fully address the specific concerns and individual needs of diverse populations, necessitating critical reflection on intervention structures to balance structure with adaptability [64].

Some study participants revealed that some therapy content did not match their concerns and needs, necessitating adjustments to ensure positive treatment outcomes and therapy adherence. Before using the ErgoLoCo program, occupational therapists had reservations about the standardized approach, fearing it might not cater to participants′ unique needs. However, they acknowledged the program′s advantages as a structured guideline for practice, which brought about a sense of relief. Consequently, our findings underscore the importance of evidence‐based, telehealth‐enabled occupational therapy interventions employing holistic methodologies and allowing for individualized adjustments. This may contribute to therapy quality and client commitment, which in turn may reduce the discontinuation rate [30].

### 4.2. Digital Accessibility

Participants and occupational therapists perceived the digital nature of the therapy program as user‐friendly. Both groups expressed satisfaction with the flexibility that allowed them to fit therapy into their very own schedule. These findings are consistent with a study on the acceptance of digital health applications in nonpharmacological therapies in Germany, where most therapists surveyed (88.7%, *n* = 133) appreciated the flexibility of therapy delivery [65]. The participants′ perspective on the convenience of telehealth is similar to those described in a systematic review on enabling and restricting factors in telehealth interventions for people with chronic pain [66]. Our findings emphasize the importance of easy access, equity, and minimal client care burden in interventions for conditions with fluctuating symptom severity and thus unpredictable symptom patterns [24–26].

While the program offered timesaving benefits, participants faced opportunity costs by sacrificing other activities, similar to attending therapy in an occupational therapy practice. Occupational therapists found the flexibility of telehealth advantageous for balancing professional roles with personal or caregiving roles. Remote work in therapy professions in Germany is still relatively uncommon. Still, studies showed that a combination of a home office and on‐site work is associated with higher job satisfaction and well‐being [67, 68].

Research using telehealth interventions has consistently revealed that digital literacy is crucial for the successful implementation and usage of these interventions. It permits individuals to effectively participate in and gain advantages from telemedicine [69–71]. Technical difficulties were identified as an issue in a study exploring the use of telehealth in occupational therapy [72]. While occasional technical challenges occurred in our study, the digital skillset among occupational therapists and participants seemed to be sufficient to ensure swift program use. Although our study′s participants and occupational therapists demonstrated these skills, it cannot be assumed that this applies to the general population of people affected by long COVID. Low digital literacy can prevent access to telehealth services [73]. The high dropout rate, particularly in the video group in our study, may be related to the participants′ lack of existing digital skills. Consequently, it is imperative to ascertain the digital skills of potential users of telehealth interventions to prevent technology‐related treatment dropouts and non‐utilisation of telehealth resources [74].

### 4.3. Professional Guidance and Support

Participants in both intervention groups expressed acceptability towards the program, particularly regarding intervention coherence, perceived effectiveness, and self‐efficacy. They felt empowered to develop strategies for managing symptoms throughout the day and during daily occupations. While the teletherapy group benefited from direct interaction and support from occupational therapists, participants in the video group often struggled to apply therapy content to their situations. The importance of receiving feedback from healthcare staff was also highlighted in another study evaluating an online self‐management program for persons with chronic conditions [64].

Client‐centeredness is a key component of occupational therapy interventions [65]. Earlier studies indicate that adapting therapy content independently in asynchronous video therapy requires a high level of initiative and health literacy from participants [63]. Our results confirm these findings; almost video group participants found it challenging to link therapy content to their individual situations, define realistic therapy goals, or perform incremental tasks. These participants reported difficulties in understanding some content, leading to frustration and incomplete tasks due to the lack of opportunity to clarify questions. These findings align with a mental health study showing that although patients were satisfied with both synchronous and asynchronous therapy sessions, those receiving asynchronous care expressed more concerns due to missing or delayed feedback [66].

### 4.4. Limitations of the Study

The present study utilized the TFA to explore the acceptability of the telehealth‐delivered occupational therapy program to both users and providers. However, a notable limitation of the study lies in its participant selection criteria, which only included individuals who completed the entire program and were willing to participate in an online interview. This omission of insights from individuals who dropped out may have missed valuable perspectives that could inform future improvements to enhance the program′s acceptability.

Despite this limitation, most participants who completed the intervention reported positive experiences. However, their positive feedback may be influenced by a lack of alternative structured intervention programs within general healthcare, leading to an inherent acceptability of the provided intervention. Participants expressed gratitude for any form of intervention throughout the study, potentially overestimating the intervention′s acceptability.

While all occupational therapists who guided the program and approximately 30% of the intervention completers were included, the findings may not be generalizable to all occupational therapists or individuals affected by long COVID. Given the homogeneous composition of the participants, it remains speculative if the program is acceptable for individuals with diverse cultural backgrounds.

### 4.5. Implications for Practice and Further Research

Future research should prioritize addressing the limitations identified in this study. This could involve adopting more inclusive participant selection criteria and employing methodologies that provide a deeper understanding of acceptability and feasibility across diverse participant demographics. Combining qualitative and quantitative approaches can enhance the generalizability of findings, ensuring interventions are effectively tailored to meet the needs of broader populations.

Moreover, further studies should focus on the participatory development and refinement of intervention concepts involving users and providers, particularly telehealth elements within occupational therapy processes. Critical reflection on telehealth elements within occupational therapy practice is essential for optimizing intervention effectiveness and adhering to established quality and ethical standards.

Further insights into the feasibility of telehealth‐based occupational therapy across various practice settings are required to facilitate the implementation of telehealth based on existing quality and ethics standards. These efforts will advance telehealth‐based therapy practice and research, ultimately enhancing the accessibility, effectiveness, and ethical integrity of interventions for diverse populations.

## 5. Conclusion

This study integrated the perspectives of users and providers of a telehealth‐delivered occupational therapy concept and evaluated its acceptability in two telehealth formats. While most participants and occupational therapists embraced the synchronous teletherapy format and viewed it as potentially beneficial for treating long COVID, some participants in the video group criticized the scope and absence of professional support in the asynchronous video format. These findings underscore the importance of incorporating professional support structures and, if applicable, peer support opportunities in both telehealth formats to align program content with individual occupational issues and rehabilitation needs.

Our results suggest that telehealth can be utilized in occupational therapy treatment of long COVID. Continuous occupational therapy monitoring could enable hybrid therapy options, offering cost‐effective long‐term therapeutic solutions and contributing to a broader array of multimodal care options. Implementing telehealth could enhance the accessibility and effectiveness of occupational therapy interventions for individuals affected by syndromes such as long COVID, ultimately improving their quality of life and facilitating their recovery journey. Further research and implementation efforts are necessary to optimize the integration of telehealth approaches into occupational therapy practice and to maximize their benefits for diverse client populations.

**Nomenclature**
COPM: Canadian Occupational Performance Measure
ErgoLoCo: acronym of the ErgoLoCo study—digital occupational therapy (ergotherapy) and long COVID
TFA: theoretical framework of acceptability

## Conflicts of Interest

The authors declare no conflicts of interest.

## Author Contributions

C.M. prepared the first draft and conducted recruitment and interviews. T.S. also conducted interviews. T.S. and D.S. were involved in the iterative development of the interview and moderation guides for data collection. T.S. and G.K. assisted in analyzing the data. S.H. is a native English speaker and translated all quotes. All authors revised the manuscript, provided further contributions and suggestions, and read and approved the final manuscript.

## Funding

This study was supported by the German Federal Ministry of Education and Research, FKZ 01EP2103A. We acknowledge support from the Open Access Publication Funds of Göttingen University.

## Data Availability

The data that support the findings of this study are available from the corresponding author upon reasonable request.
